# Molecular Structure, Comparative Analysis, and Phylogenetic Insights into the Complete Chloroplast Genomes of *Fissidens crispulus*

**DOI:** 10.3390/genes16091103

**Published:** 2025-09-18

**Authors:** Yun-Qi Song, Kai-Li Kang, Jin Chen, Yu-Mei Wei, You-Liang Xiang, Tao Peng

**Affiliations:** 1 School of Life Science, Guizhou Normal University, Huaxi District, Guiyang 550025, China; yunqsong@163.com (Y.-Q.S.); klkang2002@163.com (K.-L.K.); skychenjin@gznu.edu.cn (J.C.); 2 Guangxi Key Laboratory of Plant Conservation and Restoration Ecology in Karst Terrain, Guangxi Institute of Botany, Guangxi Zhuang Autonomous Region and Chinese Academy of Sciences, Guilin 541006, China; wushuang-123@163.com; 3 Research Center for Biodiversity, Guizhou Normal University, Huaxi District, Guiyang 550025, China

**Keywords:** *Fissidens crispulus*, chloroplast genomics, codon usage bias, Dicranidae, phylogeny, bryophyte genomics

## Abstract

Background/Objectives: *Fissidens crispulus* Brid. is a dioicous moss with conspicuous axillary hyaline nodules and serrulate leaf margins. It features *Neoamblyothallia*-type peristome teeth and serves as an ecologically significant model for studying adaptation in the hyperdiverse genus *Fissidens* (>440 species). Methods: In this study, the complete chloroplast genome of *F. crispulus* was sequenced and de novo assembled, enabling detailed comparative genomic, phylogenetic, and codon usage bias studies. Results: As the third fully sequenced member of Fissidentaceae, this study deciphers its 124,264–124,440 bp quadripartite genome encoding 129 genes (83 CDS, 32 tRNAs, 8 rRNAs). Repeat analysis identified 125–127 SSRs, dominated by mono-/di-nucleotide A/T repeats (>70%), and dispersed repeats predominantly forward (F) and palindromic (P) (>85%), confirming profound AT-biased composition (GC content: 28.7%). We established 7 hypervariable loci (*matK*, *ycf2*, etc.) as novel Dicranidae-wide phylogenetic markers. Codon usage exhibited significant A/U-ending preference, with 12 optimal codons (e.g., GCA, UGU, UUU) determined. Maximum likelihood analyses resolved *F. crispulus* and *F. protonematicola* as sister groups with high support value (MBP = 100%). Conclusions: This work provides the foundational cpDNA resource for *Fissidens*, filling a major gap in bryophyte chloroplast genomics and establishing a framework for resolving the genus’s infrageneric conflicts. Furthermore, it offers critical insights into bryophyte plastome evolution and enables future codon-optimized biotechnological applications.

## 1. Introduction

**Figure 1:**
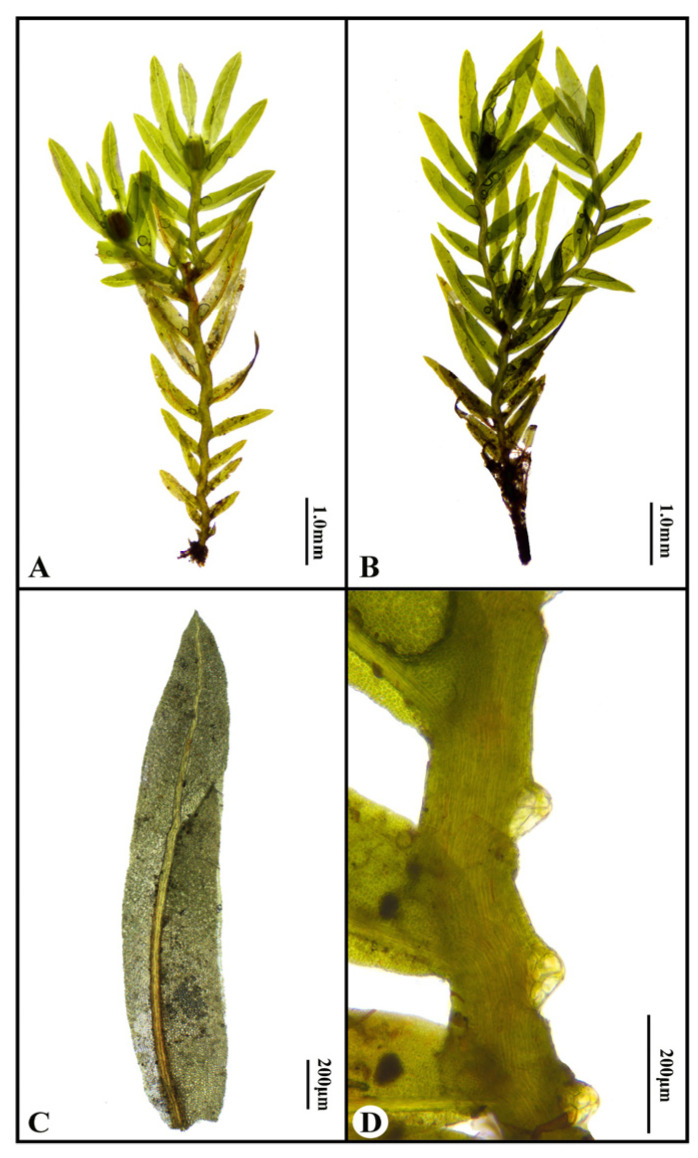
Characteristics of *Fissidens crispulus*: (**A**) the male plant; (**B**) the female plant; (**C**) the leaf. (**D**) portion of the stem showing axillary hyaline nodules.

Bryophytes constitute a major lineage within the plant kingdom, representing the second most speciose group of higher plants after seed plants [1]. They are vital components of ecosystems, playing crucial roles in water, carbon, and nitrogen cycling, as well as environmental monitoring [2,3]. Plants of the genus *Fissidens* are highly recognizable within bryophytes due to their flattened distichous leaf arrangement and distinctive differentiation of vaginant laminae, dorsal laminae, and apical laminae. This genus exhibits remarkable species diversity, with approximately 440 species distributed worldwide, primarily in tropical and subtropical regions [4,5]. Despite this diversity, genomic resources for *Fissidens* are extremely limited, with only two species having complete published genomes to date. Our study addresses this gap by presenting the third complete genome for *F. crispulus*. Based on chloroplast DNA (*rbcL* and *rps4* genes) phylogeny and morphological traits, Suzuki et al. (2018) reclassified the genus *Fissidens* into three subgenera: *Pachyfissidens*, *Neoamblyothallia*, and *Fissidens* [6]. *Fissidens crispulus* Brid. exhibits distinct morphological features. These include prominent axillary hyaline nodules, *Bryoides*-type costa in cross-section, elimbate leaf margins, and dioicous sexuality, complemented by *Neoamblyothallia*-type peristome teeth [7], see Figure 1. According to the taxonomic framework of Suzuki et al. (2018), *F. crispulus* is classified under the subgenus *Neoamblyothallia* section *Crispidium* [6].

Chloroplasts are semi-autonomous genetic organelles responsible for photosynthesis in green plants [8]. The chloroplast genome (cpDNA) of most higher plants exhibits a conserved quadripartite structure, characterized by a pair of inverted repeat regions (IRs) flanking a small single-copy region (SSC) and a large single-copy region (LSC) [9,10]. Compared to nuclear genomes, cpDNA offers distinct advantages for evolutionary studies: compact size, structural conservation, moderate evolutionary rate, high copy number, and absence of recombination. These properties make cpDNA invaluable for analyses of genetic diversity, phylogenetic reconstruction, molecular ecology, and population-level studies [11,12,13,14]. The advent of high-throughput sequencing has further revolutionized cpDNA research by enabling cost-effective genome assembly. Notably, while two complete chloroplast DNA (cpDNA) sequences of *Fissidens* are currently available in NCBI—*Fissidens nobilis* (NC044155; dioicous sexuality and *Pachyfissidens*-type of peristome teeth, classified under subgenus *Pachyfissidens* by Suzuki et al. (2018)) and *Fissidens protonematicola* (LC761303; rhizautoicous sexuality and *Fissidens*-type of peristome teeth, belonging to subgenus *Fissidens* following Suzuki et al. (2018)). This highlights a critical knowledge gap in chloroplast genomics for this diverse moss genus, as the majority of its species remain uncharacterized at the genomic level [6,7].

This study employs next-generation sequencing (NGS) platforms to sequence and conduct the first comprehensive characterization of the chloroplast genome (cpDNA) of *Fissidens crispulus* Brid. Our research objectives are to (1) resolve the structural architecture of *F. crispulus* cpDNA; (2) identify hypervariable loci across Dicranidae species; (3) detect simple sequence repeats (SSRs) and dispersed repeats; (4) reconstruct a molecular phylogenetic framework using cpDNA sequences; and (5) analyze codon usage bias (CUB) and its evolutionary determinants. These findings will provide critical resources for species delimitation, taxonomic revision, and phylogenomic resolution within *Fissidens*, advancing exploration and utilization of this ecologically significant moss lineage.

## 2. Materials and Methods

### 2.1. Data Acquisition

#### 2.1.1. Sampling, DNA Extraction, and Sequencing

Two specimens of *Fissidens crispulus* were collected from the Wangle Protection Station, Shiwandashan Mountains National Nature Reserve, Shangsi County, Guangxi Zhuang Autonomous Region, China, on 9–10 July 2024. Specimen I (Code: 20240709-12): collected on 9 July 2024 at 21°50′33.41″ N, 107°48′14.42″ E; altitude 636.6 m. Specimen II (Code: 20240710-4): collected on 10 July 2024 at 21°50′37.15″ N, 107°45′53.20″E; altitude 432.1 m. Collections were carried out by Tang Qiming and Kang Kaili. Voucher specimens were deposited in the Guangxi Institute of Botany (IBK), Guilin, China.

Total DNA was extracted via the cetyltrimethylammonium bromide (CTAB) method [15]. Subsequently, a 300–400 bp small-fragment DNA library was constructed using the DNBSEQ platform. Following library quality inspection, paired-end sequencing with a read length of 150 bp was performed on the BGISEQ platform. After sequencing, raw data were filtered using SOAPnuke to obtain high-quality clean reads, which were stored in FASTQ format [16] for downstream genome assembly and annotation.

#### 2.1.2. Chloroplast Genome Assembly and Annotation

The chloroplast genome of *Fissidens crispulus* was de novo assembled from sequencing reads using GetOrganelle v1.7.6.1 [17]. Annotation was performed by integrating results from three independent tools: CpGAVAS2 (http://47.96.249.172:16019/analyzer/annotate, accessed on 8 September 2024) [18], GeSeq [19], and Geneious Prime v2024.0.5 [20]. The annotations derived from these methods were visually compared and manually curated within Geneious Prime v2024.0.5, and then the annotated complete chloroplast genome sequence was generated. Chloroplast genome data were submitted to GenBank, with accession numbers PX108640 (*F. crispulus* I) and PX108641 (*F. crispulus* II).

Following annotation of the chloroplast genomes from two *Fissidens crispulus* individuals, key genomic features—including total sequence length, lengths of the four structural regions (LSC, SSC, IRa, and IRb), and GC content—were quantified and documented using Geneious Prime v2024.0.5. Additionally, circular genome maps for both chloroplast genomes were generated with the online tool OGDRAW [21], and their structural characteristics were systematically summarized.

### 2.2. Genome Feature Analyses

#### 2.2.1. Analysis of Inverted Repeat (IR) Boundary

Comparative analysis of inverted repeat (IR) and single-copy (SC) boundary variations between *Fissidens crispulus* and seven additional Dicranidae species was performed using IRscope (https://irscope.shinyapps.io/irapp/, accessed on 20 September 2024). The complete chloroplast genome sequences of the following species were retrieved from NCBI GenBank: *Dicranum hengduanensis* W.Z.Huang & R.L.Zhu. (OQ401775), *Fissidens nobilis* Griff. (MK876184), *Fissidens protonematicola* Sakurai (LC761303), *Leucobryum juniperoideum* (Brid.) Muell.Hal. (MK952779), *Streblotrichum convolutum* (Sm.) Lindb. (LC747010), *Syntrichia filaris* (Müll.Hal.) R.H.Zander. (MK852705), *Tortula atrovirens* (Sm.) Lindb. (PP190927), and *Weissia exserta* (Broth.) P.C.Chen (LC769575).

#### 2.2.2. Repeat Analysis

Simple sequence repeat (SSR) loci were identified using the web-based tool MISA-web (https://webblast.ipk-gatersleben.de/misa/, accessed on 20 September 2024) with the following parameters: mononucleotide SSRs (≥10 repeats), dinucleotide SSRs (≥6 repeats), trinucleotide SSRs (≥4 repeats), and tetra-, penta-, and hexanucleotide SSRs (≥3 repeats).

Dispersed repeats were analyzed in the chloroplast genomes of *Fissidens crispulus* using the REPuter online tool (https://bibiserv.cebitec.uni-bielefeld.de/reputer, accessed on 20 September 2024) with the following parameters: a Hamming distance of 3, a minimum repeat size of 30 bp, no maximum repeat size limit, and repeat types including forward, reverse, complement, and palindromic repeats.

#### 2.2.3. Nucleotide Diversity (Pi) Analysis

Consensus genes (64 genes) were extracted from 10 chloroplast genome sequences in Dicranidae using PhyloSuite v1.2.2 [22], aligned with MAFFT [23], and subjected to nucleotide diversity (Pi) analysis in DnaSP v6.1 [24] with a step size of 25 bp and a window size of the length of the associated gene. The Pi values of individual genes were subsequently visualized as a line chart in Microsoft Excel 365, and highly variable genes with Pi > 0.08 were identified for further analysis. To ensure methodological comparability, the identical set of species utilized in inverted repeat (IR) analysis was also adopted for nucleotide diversity (Pi) analysis.

### 2.3. Phylogenetic Inference

#### Phylogenetic Analysis

A phylogenetic tree of 24 species (Table S1) in the Bryopsida was established, in which the annotated *Fissidens crispulus* (‌I and II) chloroplast genomes were assembled, and the genome sequences of 23 other Bryopsida species were downloaded from NCBI, with *Sphagnum riparium*, *Sphagnum multifibrosum*, *Sphagnum junghuhnianum*, *Sphagnum subsecundum*, and *Takakia lepidozioides* as outgroups. Whole-genome sequences were aligned through MAFFT in PhyloSuite v1.2.3, and the best model GTR+F+I+G4 was selected according to Bayesian Information Criterion (BIC) in IQ-TREE [25] in PhyloSuite v1.2.3. Maximum likelihood (ML) trees were generated with 1000 bootstrap replicates using IQ-TREE in PhyloSuite v1.2.3, and the final trees were visualized and edited through the iTOL website (https://itol.embl.de/, accessed on 25 April 2025) and Adobe Illustrator v2025.

### 2.4. Codon Usage Bias Analyses

#### 2.4.1. Calculation of Parameters Related to Codon Usage Bias

Using Geneious Prime v2024.0.5, we extracted annotated coding sequences (CDSs) >300 bp that fulfilled these criteria: initiation by an ATG start codon, termination with standard stop codons (TAA, TAG, or TGA), absence of in-frame stop codons, and exclusion of chloroplast genome duplicates (i.e., duplicated genes in the IR regions). In the *Fissidens crispulus* chloroplast, 53 (I) and 54 (II) protein-coding genes were subjected to codon preference analysis.

Using CodonW v1.4.2 [26], we analyzed the GC content at the third position of synonymous codons (GC3s). Additionally, the Genepioneer online software (http://cloud.genepioneer.com/, accessed on 20 January 2025) was used to analyze and tally CDS sequence length, and the A, T, G, and C content at the third position of each codon (A3, T3, G3, and C3), as well as the GC content at the first (GC_1_), second (GC2), and third (GC3) positions of the codons, and the overall GC content (GCall).

Pearson correlation was conducted and heatmaps were generated using OriginPro v2025b [27] to examine the relationships among the effective number of codons (ENC), total GC content (GCall), GC content at the first (GC1), second (GC2), and third (GC3) codon positions, and CDS sequence length.

#### 2.4.2. Neutrality Plot Analysis

Neutrality plot analysis was performed by plotting GC12 (the average of GC1 and GC2) on the *y*-axis against GC3 on the *x*-axis, with each data point representing a specific gene [28]. The plot incorporated a trendline, the diagonal (y = x), and the coefficient of determination (R^2^) to evaluate the relationship between variables. When GC12 and GC3 exhibited a significant correlation (indicated by a high R^2^ value), it suggested that codon usage bias was predominantly influenced by mutational pressure. Conversely, if the correlation was weak (low R^2^) and data points were primarily distributed above the diagonal, this signified that natural selection was the dominant factor shaping codon usage patterns [29].

#### 2.4.3. ENC-Plot Analysis

ENC-plot analysis was performed by constructing a two-dimensional scatter plot with GC3s content plotted on the *x*-axis and ENC value plotted on the *y*-axis. The theoretical expected curve, reflecting codon usage bias determined solely by base mutation pressure, was incorporated for analysis. The formula defining this standard curve is ENC = 2 + GC_3_ + 29/[GC_3_^2^ + (1 − GC_3_)^2^]. By comparing the distribution characteristics of the actual data points relative to this theoretical curve, the relative contributions of natural selection pressure and mutational bias to codon usage bias can be effectively evaluated [30]. Genes whose data points lie on or near the standard curve indicate that their codon usage bias is predominantly governed by mutational pressure. Conversely, a distribution showing significant deviation from the standard curve suggests that natural selection is the primary influencing factor.

#### 2.4.4. Parity Rule 2 (PR2) Bias Plot Analysis

PR2-plot analysis was performed by constructing a scatter plot with G3/(G3 + C3) plotted on the *x*-axis and A3/(A3 + T3) plotted on the *y*-axis. The central point (0.5, 0.5) of this plot represents the state of codon usage in the absence of bias, where A = T and C = G [31]. The vector distance between each gene’s data point and this theoretical center point (0.5, 0.5) reflects the magnitude of bias, while the direction of displacement (e.g., toward G/C or A/T) reveals nucleotide-specific preferences [32]. By examining the distribution pattern within the scatter plot and its characteristic deviation relative to the central point, key factors influencing codon usage bias can be further analyzed.

#### 2.4.5. Relative Synonymous Codon Usage (RSCU) Analysis and Identification of Optimal Codons

The relative synonymous codon usage (RSCU) analysis was performed using CodonW v1.4.2 to calculate RSCU frequency for codons corresponding to each amino acid. The resulting data were visualized as a heatmap using Genepioneer online software.

For optimal codon identification, ENC of chloroplast genes served as the metric for codon usage bias analysis. All genes were screened and sorted in ascending order based on ENC values. The top 10% (low ENC) and bottom 10% (high ENC) genes from this sorted list were selected to construct high-expression and low-expression gene pools, respectively. Using CodonW v1.4.2, RSCU values for codons within each pool were computed. High-expression codons were identified by comparing RSCU differences between pools (ΔRSCU = RSCU_high_ − RSCU_low_), with codons satisfying ΔRSCU ≥ 0.08 designated as such. Concurrently, codons with RSCU > 1 were defined as high-frequency codons. Codons meeting both criteria—high-frequency (RSCU > 1) and high-expression (ΔRSCU ≥ 0.08)—were identified as optimal codons [33,34].

## 3. Results

### 3.1. Chloroplast Genome Characteristics of Fissidens crispulus

**Figure 2:**
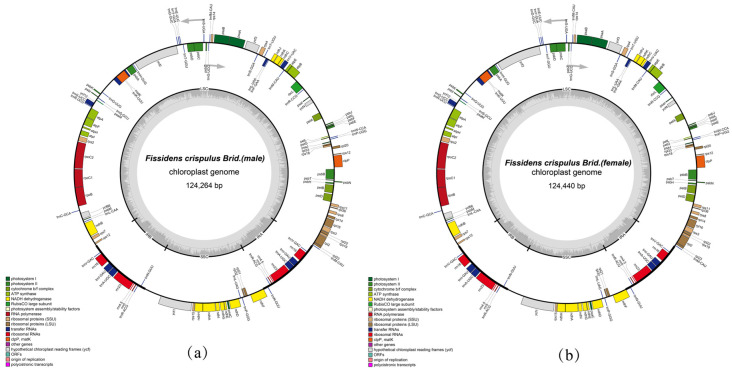
Circular maps of the chloroplast genomes for *Fissidens crispulus*. The large single-copy (LSC) and small single-copy (SSC) regions are separated by a pair of inverted repeats (IRa and IRb). Genes drawn inside the circle are transcribed clockwise, while those on the outside are transcribed counterclockwise. Genes are colored according to their functional categories: (**a**) *F. crispulus* I; (**b**) *F. crispulus* II.

The chloroplast genomes of *Fissidens crispulus* (I and II) exhibit a typical quadripartite structure, with a total length, 124,264 –124,440 bp, respectively. It comprises a small single-copy region (SSC) of 18,608–18,609 bp, a large single-copy region (LSC) of 85,572–85,731 bp, and a pair of inverted repeat regions (IR), each 10,042–10,050 bp (Table 1, Figure 2).

The chloroplast genome of *Fissidens crispulus* is annotated with 129 genes, including 83 coding sequences (CDS), 32 transfer RNA (tRNA) genes, and 8 ribosomal RNA (rRNA) genes (Table S2, Figure 2). Among these, 65 genes are associated with self-replication: 10 are linked to the ribosomal large subunit, and 11 to the ribosomal small subunit. A total of 47 genes participate in photosynthesis, including 6 ATP synthase genes, 11 NADH dehydrogenase genes, 5 cytochrome complex-related genes, 6 photosystem I genes, 15 photosystem II genes, 1 Rubisco-related gene, and 3 protochlorophyllide reductase-related genes (Table S2, Figure 2). Additionally, 12 genes are annotated for other functions (e.g., *accD*, *cemA*, *ccsA*, *clpP*, *matK*, *infA*) or unknown roles (e.g., *ycf1*, *ycf2*, *ycf3*, *ycf4*, *ycf12*, *ycf66*) (Table S1, Figure 2). Seventeen genes contain a single intron (*ndhA*, *ndhB*, *petB*, *petD*, *atpF*, *rpl16*, *rpl2*, *rpoC1*, *ycf66*, *trnA-UGC*, *trnI-GAU*, *trnK-UUU*, *trnL-UAA*, *trnV-UAC*, and *trnG-UCC*), while *clpP* and *ycf3* each possess two introns (Table S2).

### 3.2. IR Expansion and Contraction in Chloroplast Genomes

**Figure 3:**
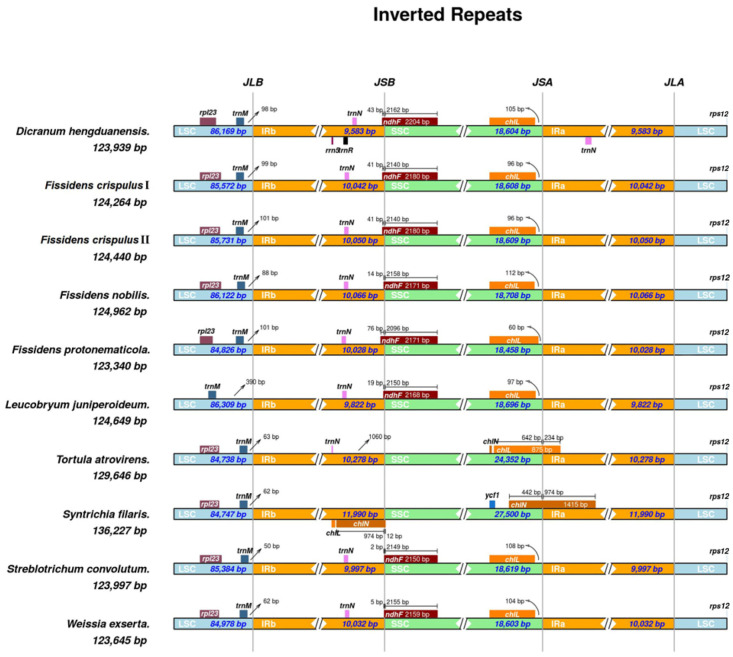
Comparison of the large single-copy (LSC) region, small single-copy (SSC) region, and inverted repeat (IR) junctions among the chloroplast genome sequences of nine Dicranales species.

Structural analysis of the inverted repeat (IR) boundary regions in chloroplast genomes revealed variations in gene composition and length across junction sites (Figure 3). Among the nine Dicranidae species, the size ranges were 84,738–86,169 bp for the large single-copy (LSC) region, 18,458–27,500 bp for the small single-copy (SSC) region, and 9583–11,990 bp for the inverted repeat (IR) regions. *Tortula atrovirens* showed the smallest LSC region (84,738 bp), while *Dicranum hengduanensis* had the largest (86,169 bp). The SSC region was shortest in *Fissidens protonematicola* (18,458 bp) and longest in *Syntrichia filaris* (27,500 bp). The IR regions were smallest in *Dicranum hengduanensis* (9583 bp) and largest in *Syntrichia filaris* (11,990 bp).

At the JLB (IRb/LSC) boundary, *rpl23* was absent from the large single-copy (LSC)-proximal region in *Leucobryum juniperoideum*. In the eight other species examined, both *rpl23* and *trnM* were positioned in the LSC. For *trnM* of the nine species, the distance from the JLB boundary ranged from 62 to 390 bp (minimum 62 bp in *Syntrichia filaris*, maximum 390 bp in *Leucobryum juniperoideum*).

At the JSB (IRb/SSC) boundary, *trnN* occurred on the left in all species except *Syntrichia filaris*. On the right, *ndhF* crossed the boundary in all species except *Syntrichia filaris* and *Tortula atrovirens*, extending into the IRB region by 2–76 bp (minimum 2 bp in *Streblotrichum convolutum*, maximum 76 bp in *Fissidens protonematicola*).

At the JSA (IRa/SSC) boundary, *chlL* was present in all eight species except *Syntrichia filaris*, which possessed *chlN* instead. In *Tortula atrovirens*, *chlL* straddled the JSA boundary. In contrast, the gene was confined to the SSC region in the other seven species, with distances from the JSA boundary ranging from 60 to 112 bp (*Fissidens protonematicola*: minimum 60 bp; *Fissidens nobilis*: maximum 112 bp).

Comparative analysis revealed complete collinearity of the *F. crispulus* plastome with those of *F. polymnia* and *F. taxifolius*, with no large-scale inversions or translocations detected, indicating strong structural conservation within the genus.

### 3.3. Repeat Sequence Analysis

**Figure 4:**
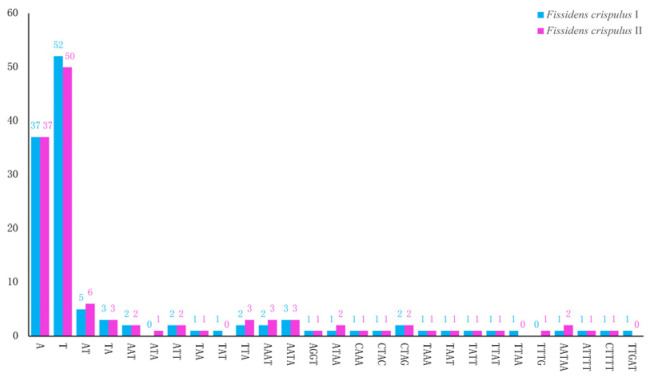
Numbers and types of SSR in the chloroplast genome of *Fissidens crispulus*. The *X*-axis represents types of SSR, while the *Y*-axis represents the number of SSR.

The chloroplast genomes of *Fissidens crispulus* I and II contained 125 and 127 SSR loci, respectively. Mononucleotide repeats (A/T only) dominated the SSRs in both samples (*F. crispulus* I: 37A, 52T; *F. crispulus* II: 37A, 50T), consistent with the AT-rich nature of chloroplast DNA. Dinucleotide repeats were exclusively AT/TA (*F. crispulus* I: 5AT, 3TA; *F. crispulus* II: 6AT, 3TA). Tri-, tetra-, and pentanucleotide repeats were less frequent but present in both samples (Figure 4).

**Figure 5:**
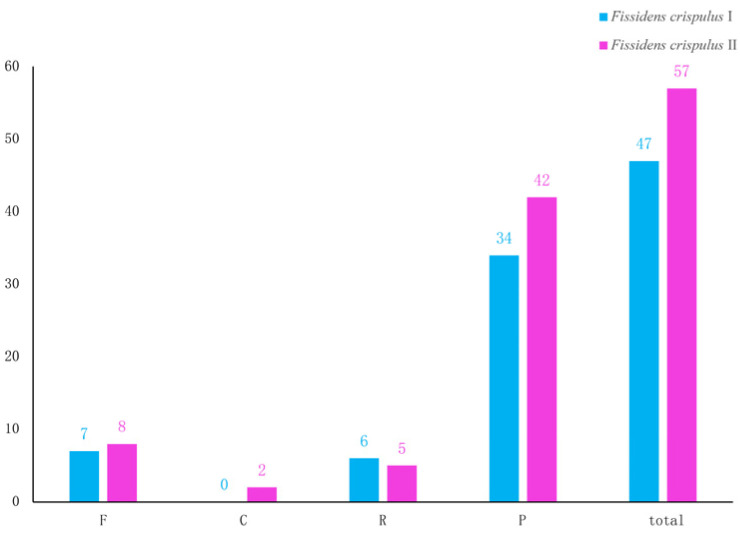
Numbers and types of interspersed repeats in the chloroplast genome of *Fissidens crispulus*. Forward repetition is abbreviated as F, palindromic repetition is abbreviated as P, reverse repetition is abbreviated as R, and complementary repetition is abbreviated as C. The *X*-axis represents types of interspersed repeats, while the *Y*-axis represents the number of interspersed repeats.

For interspersed Repeats, *F. crispulus* I and II possessed 47 and 57 interspersed repeats, respectively. Palindromic (P-type) repeats constituted the most abundant category in both genomes (*F. crispulus* I: 34; *F. crispulus* II: 42). Forward (F-type), reverse (R-type), and complementary (C-type) repeats were less common (Figure 5).

### 3.4. Nucleotide Diversity Analysis

**Figure 6:**
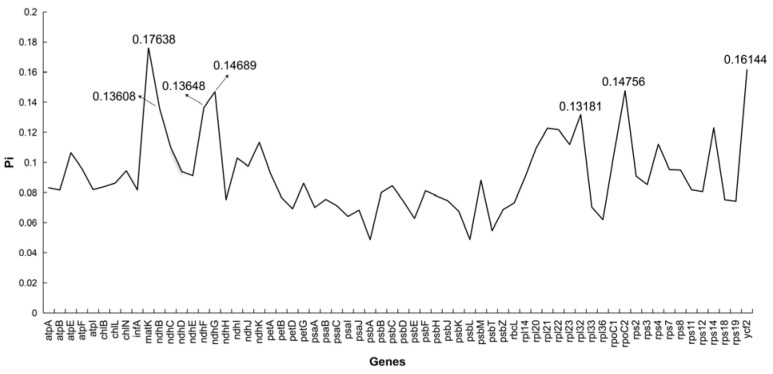
Nucleotide polymorphism analysis of the chloroplast genomes of Dicranidae species. The *X*-axis represents genes, while the *Y*-axis represents nucleotide diversity values.

Nucleotide diversity (Pi) analysis was conducted on nine species within the Dicranidae. Statistical results (Figure 6) revealed that the Pi values ranged from 0.04882 to 0.17638, with an average of 0.09197. Among these, the top seven genes with Pi values are *matK*, *ycf2*, *rpoC2*, *ndhG*, *ndhF*, *ndhB*, and *rpl32*. The identification of these hypervariable regions provides a foundation for designing targeted primers or barcodes to enhance resolution in species delimitation and phylogenomic analyses.

### 3.5. Phylogenetic Analysis

**Figure 7:**
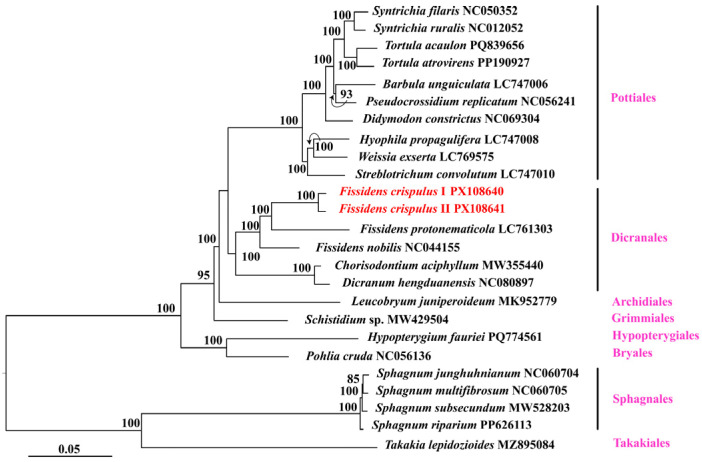
Phylogenetic tree inferred by the maximum likelihood (ML) method based on 24 representative species. A total of 1000 bootstrap replicates were computed. The maximum bootstrap percentages (MBPs) are shown at the branches, and branch lengths are shown by the scale.

Within Dicranales, phylogenetic analysis strongly supported (MBP = 100%) the monophyly of *Fissidens crispulus*, represented by accessions I and II. This *F. crispulus* clade was strongly resolved as sister (MBP = 100%) to *Fissidens protonematicola*. The clade comprising *F. crispulus* and *F. protonematicola* was further strongly supported as sister (MBP = 100%) to *Fissidens nobilis*. Furthermore, this *Fissidens* clade (*F. crispulus*, *F. protonematicola*, *F. nobilis*) was robustly resolved as sister (MBP = 100%) to the clade comprising *Chorisodontium aciphyllum* and *Dicranum hengduanensis*. Dicranales showed a weakly supported sister relationship (MBP < 50%) with Pottiales. However, the Dicranales-Pottiales clade itself formed a strongly supported sister group (MBP = 100%) with Archidiales (Figure 7).

### 3.6. Correlation Analysis of Codon Usage Parameters

**Figure 8:**
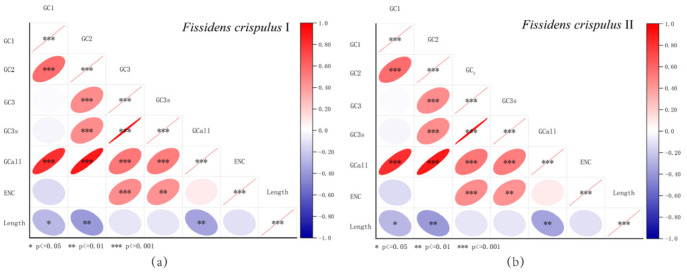
Correlation analysis in *Fissidens crispulus*. The correlations among codon base composition (GC1, GC2, GC3, GC3s, and GCall), effective number of codons (ENC), and length of genes (Length) were analyzed in *F. crispulus*, respectively. The color of the color block changes from blue to red, indicating that the correlation is increasing. One asterisk (*) indicates a significant correlation among indices at *p* ≤ 0.05; two asterisks (**) indicate the correlation at *p* ≤ 0.01; three asterisks (***) indicate the correlation at *p* ≤ 0.001: (**a**) *F. crispulus* I; (**b**) *F. crispulus* II.

Correlation analyses of GC1, GC2, GC3, GC3s, GCall, and ENC parameters in *Fissidens crispulus* chloroplast genomes are summarized in Table 2 and visualized through a correlation heatmap (Figure 8). Significant correlations were observed between GC and GC1, GC2, and GC3 (all *p* < 0.01), indicating coordinated compositional trends across codon positions. GC1 and GC2 exhibited a highly significant correlation (*p* < 0.01), as did GC2 and GC3 (*p* < 0.01), suggesting similarities in base composition between the first and second codon positions, as well as between the second and third positions.

The ENC value showed no significant correlation with GC1 or GC2 but demonstrated a strong negative correlation with GC3 (*p* < 0.01), highlighting the dominant role of third-position base composition in shaping codon usage bias (CUB). Notably, ENC exhibited no significant association with gene length (Length), confirming that sequence length had minimal impact on codon bias analysis and ruling out potential artifacts from short gene sequences.

### 3.7. Codon Usage Bias Analyses

#### 3.7.1. Neutrality Plot Analysis

**Figure 9:**
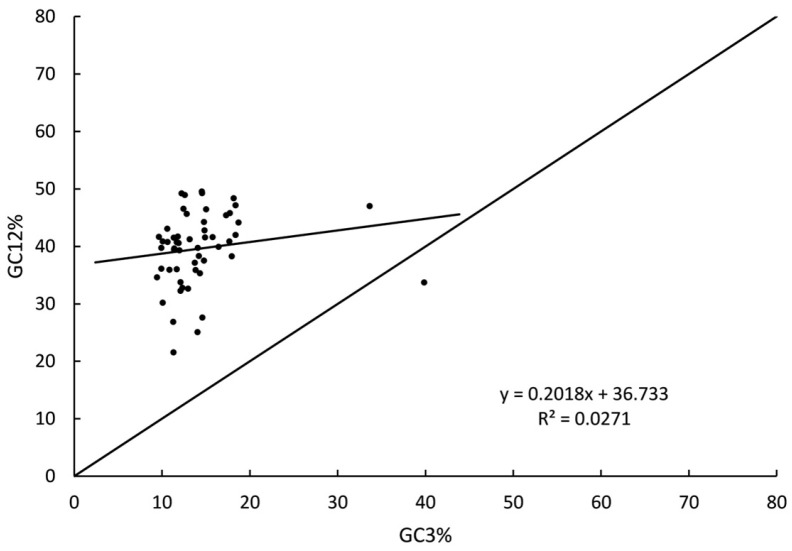
Neutrality plot analysis in *Fissidens crispulus* I. The correlations between the average GC codon content in GC1 and GC2 (GC12) and the third codon position (GC3) were analyzed, and the standard curve and R^2^ in *F. crispulus* I, respectively. The *X*-axis represents GC3%, while the *Y*-axis represents GC12%.

The neutrality plot results (Figure 9) revealed that the majority of chloroplast genes in *Fissidens crispulus* I were distributed above the diagonal line, with only one gene (*rps12*) located below the diagonal. Most genes were positioned far from the diagonal. The regression coefficient was 0.2018, while the squared correlation coefficient (*r*^2^) was 0.0271. Two-tailed tests indicated no significant correlation (*p* > 0.05) across the dataset. An identical pattern was observed for *F. crispulus* II (data presented in Figure S1).

#### 3.7.2. ENC-Plot Analysis

**Figure 10:**
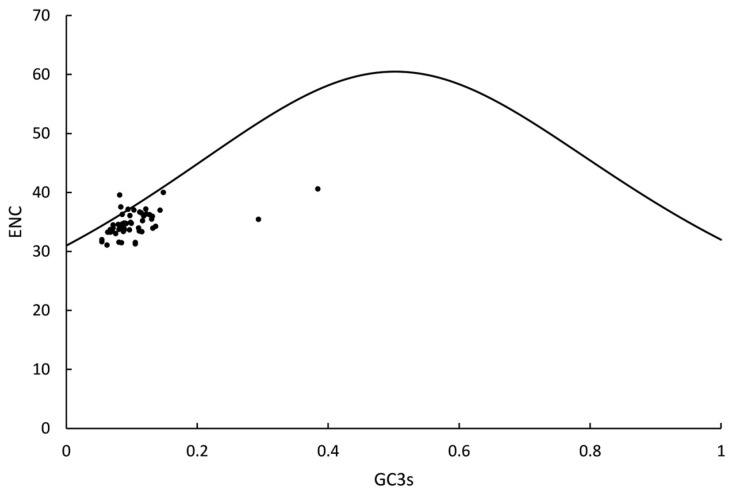
ENC-GC3 plot analysis in *Fissidens crispulus* I. The correlations between the effective number of codons (ENC) and the contents of the nucleotide G/C at the third codon synonymous location (GC3s) were analyzed in *F. crispulus* I, respectively. The standard curve represents the functional relationship between ENC and GC3 under mutation pressure rather than natural selection. The *X*-axis represents GC3s, while the *Y*-axis represents ENC.

As illustrated by the ENC-plot analysis (Figure 10), the majority of genes in *Fissidens crispulus* exhibited ENC values ranging between 30 and 40. All protein-coding genes displayed ENC values above 30, indicating relatively weak codon usage bias (CUB) in the chloroplast genomes of this species. Furthermore, the distribution of most genes is closely aligned with the standard ENC-GC3s curve. For *F. crispulus* II, a consistent pattern was detected, with supporting data outlined in Figure S2.

#### 3.7.3. Parity-Rule 2 (PR2) Bias Plot Analysis

**Figure 11:**
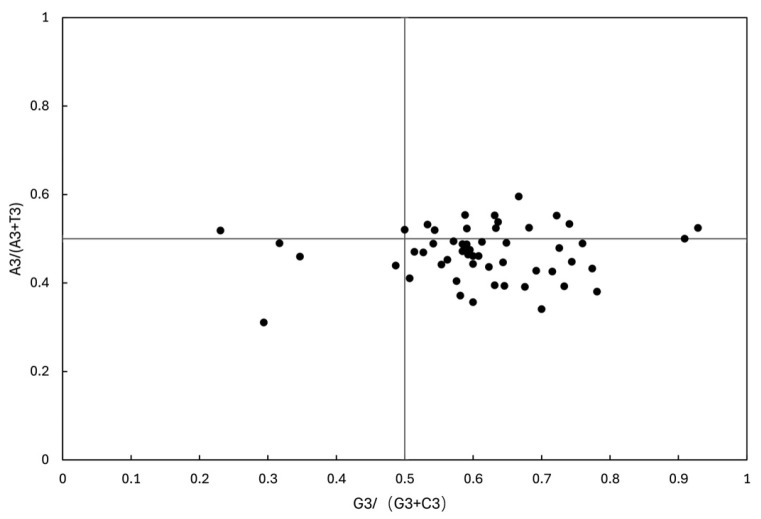
PR2-bias plot analysis in *Fissidens crispulus* I. The correlations between A3/(A3 + U3) and G3/(G3 + C3) were analyzed in *F. crispulus* I, respectively. If the codon has no usage bias, A = T and C = G, the value is at the center point of the plot. The first quadrant represents the codon preference of A/G, and the third quadrant represents T/C preference. The *X*-axis represents G3/(G3+C3), while the *Y*-axis represents A3/(A3 + U3).

The PR2-plot analysis (Figure 11) revealed an asymmetric distribution of data points across the four quadrants, with a predominant concentration in the region where G3/(G3 + C3) > 0.5 and A3/(A3 + T3) < 0.5, indicating a significant bias favoring thymine (T) over adenine (A) and guanine (G) over cytosine (C) at the third codon position. A congruent pattern was observed in *Fissidens crispulus* II, with supplementary data detailed in Figure S3.

#### 3.7.4. Analysis of RSCU and Optimal Codons

**Figure 12:**
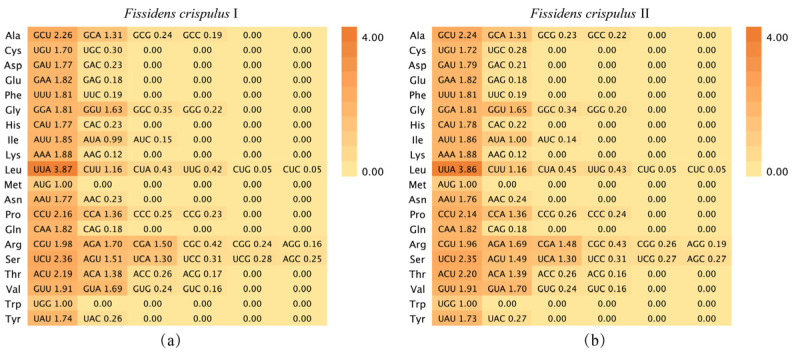
Relative synonymous codon usage (RSCU) value was calculated by dividing the amino acids encoded by the same codons and their probability of appearing in the same codons. The color of the color block changes from light to dark, indicating that the RSCU values are increasing, of which an RSCU value > 1 indicates a positive codon bias: (**a**) *Fissidens crispulus* I; (**b**) *F. crispulus* II.

As shown in Figure 12, *Fissidens crispulus* exhibited 28 codons with RSCU values > 1, accounting for 45.90% of the total 61 codons (classified as high-frequency codons). Among these high-frequency codons, the number of those ending with A ranged from 11 to 12 (corresponding to 39.29–42.86% of the high-frequency codons), while codons ending with U were consistently 16 (representing 57.14%), with no codons ending in G or C. As a result, the proportion of high-frequency codons terminating in A or U spanned from 96.43% to 100%. This highlights the overwhelming dominance of A- and U-ending codons in the chloroplast genome of *F. crispulus*.

As shown in Table 3, shared optimal codons between the two samples included GCA, UGU, UUU, GGU, UUA, AAU, CCU, CGA, CGU, AGU, UCA, and ACU. I-specific codons were AUG, ACA, and UGG, while II-specific ones were GCU, AUU, UCU, and GUU. In *Fissidens crispulus*, U-terminated codons dominated the optimal codon repertoire, underscoring their evolutionary significance in chloroplast genome translation.

## 4. Discussion

### 4.1. Chloroplast Features of Fissidens crispulus

In this study, two chloroplast genome sequences of *Fissidens crispulus* were obtained, providing a fundamental basis for genetic engineering, phylogenetic studies, and species identification of this species. The chloroplast genome of *F. crispulus* exhibits the typical IR-SC quadripartite structure of higher plant chloroplasts, consisting of two inverted repeat (IR) regions, one large single-copy (LSC) region, and one small single-copy (SSC) region. Its gene content, gene order, and GC content are consistent with those of most species in the subclass Dicranidae [35,36,37], indicating a high degree of structural conservation in the chloroplast genome of *F. crispulus*.

The contraction and expansion of boundaries between IR (inverted repeat) and SC (single-copy) regions are key structural variation factors influencing chloroplast genome size [38]. In this study, chloroplast genomes of nine Dicranidae species were analyzed to elucidate gene distribution patterns across the IR-SC boundary regions. Specifically, both genomes of *Fissidens crispulus* showed high similarity with the congeneric *Fissidens nobilis* and *Fissidens protonematicola* in gene categories, gene positions, and lengths of chloroplast genome regions; moreover, no obvious differences were observed when compared with species of other genera. Although the chloroplast genomes of the two sequenced specimens were identical, suggesting local genome conservation, further sampling across the species’ distribution range (e.g., China, Japan, and Korea) is needed to assess potential geographic variation. Comparative analyses of multiple populations would clarify whether intraspecific plastome divergence exists and help elucidate the phylogeographic history of *F. crispulus*. These structural patterns align with comparative analyses revealing marked structural conservation in both inverted repeat (IR) and single-copy (SC) regions across the clade, consistent with the genomic stability observed in other bryophytes such as *Sphagnum* species [39]. However, *Syntrichia filaris* and *Tortula atrovirens* emerged as exceptions, exhibiting minimal IR contraction compared to other bryophyte species, which resulted in distinct gene arrangements at their JSA (IRA/SSC boundary) and JSB (IRB/LSC boundary).

Genomic repetitive sequences, especially SSRs (simple sequence repeats), are important clues for deciphering species’ evolutionary history and genetic differentiation. In the chloroplast genome of *Fissidens crispulus*, A/T mononucleotide repeats dominate SSRs, which is consistent with the general understanding that *“chloroplast SSRs are mainly composed of A/T with low proportions of C/G repeats”* [40]. The preference for A/T-related sequence features may be driven by mutation pressure: A/T base pairs have fewer hydrogen bonds than G/C pairs, making them more prone to substitution during DNA replication or repair, and this low-energy mutation characteristic may lead to the formation of such preferences [41,42]. The prevalence of A/T-rich SSRs in the *F. crispulus* plastome is not merely a confirmation of a known trend but has significant broader implications. This underscores that the mutational pressures driving chloroplast evolution are deeply conserved across land plants, including bryophytes. For applied science, this compositional bias provides a blueprint for the effective design of synthetic genetic circuits for chloroplast engineering and identifies native hypervariable regions that are premier candidates for developing high-resolution molecular markers for population genetics and species barcoding within the Bryophyta.

Based on nucleotide diversity (Pi) analysis, the top seven genes ranked by nucleotide diversity (Pi values) are *matK*, *ycf2*, *rpoC2*, *ndhG*, *ndhF*, *ndhB*, and *rpl32*; thus, these 7 genes are highly variable coding genes in the chloroplast genome of Dicranidae. By comparing all coding sequences and calculating Pi values for 64 protein-coding genes based on chloroplast genome data, 7 highly variable coding genes were identified. These hypervariable loci offer significant potential for taxonomic and conservation applications. Their high mutation rates make them ideal candidate molecular markers for resolving phylogenetic relationships at the order level within Dicranidae, where traditional morphological traits may be insufficient or convergent. In taxonomic studies, these genes can help clarify species boundaries, identify cryptic diversity, and reconstruct robust phylogenies, thereby improving classification accuracy. For conservation, such variable markers facilitate the assessment of genetic diversity within and among populations, support the identification of evolutionarily significant units (ESUs), and help prioritize populations for conservation based on their genetic distinctiveness or adaptive potential. Thus, these genes provide valuable tools for enhancing both systematic research and biodiversity conservation strategies.

### 4.2. Phylogenetic Relationships of Fissidens crispulus

In our study, *Fissidens crispulus* and *F. protonematicola* share a closer phylogenetic relationship to each other than to *Fissidens nobilis*, a finding consistent with previous studies [6,43,44]. The weak support for the Dicranales-Pottiales clade underscores the need for expanded taxon sampling to robustly resolve these deep relationships and fully understand the early evolutionary radiation of the Bryopsida. Broader phylogenetic positions of Dicranales, Archidiales, Grimmiales, Hypopterygiales, Bryales, Sphagnales, and Takakiales aligned with established chloroplast genome-based frameworks for moss evolution [45,46,47,48].

### 4.3. Natural Selection Plays a Critical Role in Shaping Codon Usage Bias in Fissidens crispulus

Neutrality plot (Figure 9) indicated distinct mutation patterns between the first/second positions (GC12) and the third position (GC3) of codons in the chloroplast genome of *Fissidens crispulus*. Notably, GC12 exhibited a lower degree of neutral mutation compared to GC3, reflecting significant divergence in their evolutionary patterns. The codon usage bias (CUB) was predominantly influenced by natural selection rather than base mutation.

From the ENC-plot (Figure 10), the distribution of most genes closely aligned with the standard ENC-GC3s curve, implying that natural selection, rather than mutation pressure, predominantly governs the codon usage preferences in *Fissidens crispulus*. The limited deviation of data points from the theoretical neutral evolution trajectory underscores the dominant role of selective constraints in shaping chloroplast genome codon optimization.

According to neutral theory, if codon usage bias (CUB) were solely driven by mutational pressure, parity in A/T and G/C ratios would be expected. From the PR2-plot (Figure 11), the observed deviation demonstrates that the codon usage patterns in the chloroplast genome of *Fissidens crispulus* are shaped by both mutational pressure and natural selection, with the latter exerting a stronger influence.

Based on the neutral theory of molecular evolution, codon usage bias (CUB) arises from the combined effects of evolutionary forces such as natural selection and mutation pressure [49]. In this study, codon composition analysis of the chloroplast genome of *Fissidens crispulus* revealed a significantly higher proportion of A/U-ending codons compared to G/C-ending codons (Table 3). Combined results from neutrality plot analysis (Figure 9), ENC-Plot analysis (Figure 10), and PR2-plot analysis (Figure 11) demonstrate that natural selection dominates the formation of codon usage bias in *F. crispulus*.

### 4.4. Optimal Codons Provide Novel Insights for Chloroplast Genetic Engineering in Fissidens crispulus

Codon usage bias (CUB) serves as a key metric for cross-species comparison of synonymous codon selection, providing theoretical foundations for optimizing heterologous gene expression. Similar to other plant chloroplast genomes [50,51,52,53,54], the chloroplast genome of *Fissidens crispulus* contains 28 high-frequency codons (RSCU > 1), all of which end with A/U. Based on the RSCU values of the *F. crispulus* chloroplast genome (selection criteria: RSCU > 1 and ΔRSCU ≥ 0.8), 12 shared optimal codons were identified in the two sequences, GCA, UGU, UUU, GGU, UUA, AAU, CCU, CGA, CGU, AGU, UCA, and ACU, which also exhibit a preference for A/U endings. This A/U-ending codon preference is consistent with the codon usage patterns of higher green plants, reflecting the cross-taxonomic conservation of codon usage bias in chloroplast genomes [55,56]. Moreover, the strong A/U bias may reflect an evolutionary adaptation to the shaded and moist environments where *Fissidens* species thrive, as AT-rich genomes tend to require less energy for replication and repair—a potential advantage in low-light conditions where energy availability is limited. This codon usage pattern could further enhance translational efficiency under environmental stress, supporting protein synthesis in habitats with fluctuating moisture and light levels. In plant chloroplast genomes, optimal codon selection also improves translational fidelity and efficiency through preferential pairing with abundant tRNAs, consequently elevating gene expression levels. The optimal codons identified in this study through chloroplast genome CUB analysis provide critical references for optimizing the heterologous expression efficiency of exogenous genes in moss chloroplasts. This finding particularly highlights its application value in synthetic biology: these evolutionarily conserved codon usage patterns can inform precise codon optimization strategies for chloroplast genetic transformation systems, significantly boosting the production of target proteins.

### 4.5. Future Research Directions

Building on this study, several promising research avenues emerge. First, expanding chloroplast genomic sequencing to encompass a broader range of *Fissidens* taxa—especially from under-represented biogeographic regions and morphologically cryptic species complexes—will help clarify phylogenetic relationships and refine taxonomic boundaries across the genus. Second, functional validation of the observed codon usage bias through experimental expression assays can elucidate its role in adaptive responses to environmental stressors such as low light intensity or fluctuations in water availability. Third, population-level studies utilizing the hypervariable markers identified in this work may reveal patterns of genetic diversity, gene flow, and local adaptation among geographically dispersed populations of *F. crispulus*. In addition, integrative analyses combining nuclear and mitochondrial genomic data will offer a more holistic understanding of evolutionary mechanisms in bryophytes. Finally, the potential applications of codon-optimized sequences in synthetic biology—including the development of efficient transplastomic systems for non-vascular plants—merit further experimental investigation.

## 5. Conclusions

This study reports the third complete cpDNA from *Fissidens*. Key findings include a strong A/U-ending codon bias, identification of 12 optimal codons, numerous SSRs and dispersed repeats, and seven highly variable genes. Phylogenetically, *F. crispulus* forms a fully supported clade with *F. protonematicola* and *F. nobilis*. These results provide a practical foundation for enhancing exogenous gene expression in chloroplast genetic engineering within *Fissidens* through optimized codon usage. The highly variable genes also offer valuable molecular markers for resolving taxonomic relationships. Furthermore, comprehensive analyses confirm that natural selection—not mutation—is the dominant force influencing codon usage patterns. This work establishes essential genomic resources and an evolutionary framework for future research and biotechnological applications in this genus. Looking forward, the genomic data presented here will facilitate high-resolution DNA barcoding for accurate species identification and support conservation efforts for rare *Fissidens* species. Moreover, this work provides a foundation for developing chloroplast-based genetic engineering tools specifically tailored to bryophytes. Future research should focus on functional validation of the identified genetic elements and expand comparative genomic studies across bryophytes to further elucidate chloroplast evolution and regulatory mechanisms.

## Figures and Tables

**Table 1:** Composition and characteristics of chloroplast genomes of *Fissidens crispulus*.

Species	Size (bp)	LSC (bp)	SSC (bp)	IR (bp)	Total GC (%)	LSC GC (%)	SSC GC (%)	IR GC (%)
*Fissidens crispulus* I	124,264	85,572	18,608	10,042	28.7	26.0	25.1	43.3
*Fissidens crispulus* II	124,440	85,731	18,609	10,050	28.7	26.0	25.2	43.4

**Table 2:** Correlation analysis of codon parameters in *Fissidens crispulus* chloroplast genes.

Species	Variable	Correlation Coefficient					
		GC1	GC2	GC3	GC3s	GCall	ENC
*F. crispulus* I	GC2	0.560					
	GC3	−0.061	0.436				
	GC3s	−0.121	0.369	0.963			
	GCall	0.786	0.890	0.502	0.426		
	ENC	−0.124	0.08	0.428	0.511	0.114	
	Length	−0.308	−0.425	−0.108	−0.067	−0.391	−0.052
*F. crispulus* II	GC2	0.579					
	GC3	−0.001	0.441				
	GC3s	−0.023	0.444	0.996			
	GCall	0.803	0.889	0.526	0.513		
	ENC	−0.122	0.005	0.447	0.432	0.088	
	Length	−0.279	−0.387	−0.099	−0.098	−0.351	−0.102

**Table 3:** Optimal codons in the chloroplast genome of *Fissidens crispulus*.

Category	Codons	Count	Number of U-Ending Codons (Percentage)
Shared Codons	GCA, UGU, UUU, GGU, UUA, AAU, CCU, CGA, CGU, AGU, UCA, ACU	12	8 (66.67%)
*Fissidens crispulus* I-Specific	AUG, ACA, UGG	3	0 (0%)
*Fissidens crispulus* II-Specific	GCU, AUU, UCU, GUU	4	4 (100%)
Total		19	12 (63.16%)

## Data Availability

The chloroplast genome data of *Fissidens crispulus* sequenced in this study have been deposited in the NCBI Nucleotide Database (https://www.ncbi.nlm.nih.gov/nuccore; accessed on 11 August 2025) under accession numbers PX108640 (*Fissidens crispulus* I) and PX108641 (*Fissidens crispulus* II).

## Supplementary Materials

The following supporting information can be downloaded at; https://www.mdpi.com/article/10.3390/genes16091103/s1, Table S1: Species used for constructing phylogenetic trees and their GenBank accession numbers; Table S2: Functional annotation and classification of chloroplast genomes of *Fissidens crispulus*; Figure S1: Neutrality plot analysis in *F. crispulus* II; Figure S2: ENC-GC3 plot analysis in *F. crispulus* II; Figure S3: PR2-bias plot analysis in *F. crispulus* II.
