# Supplementary material for: Molecular Structure, Comparative Analysis, and Phylogenetic Insights into the Complete Chloroplast Genomes of Fissidens crispulus
Source: Genes (Basel). 2025 Sep 18;16(9):1103. doi: 10.3390/genes16091103 (PMC12469583; doi:10.3390/genes16091103)
Supplement: Supplementary file 1 [file genes-16-01103-s001.zip › genes-3845664-supplementary.pdf]

Table S1. Species used for constructing phylogenetic trees and their GenBank accession numbers

Species	GenBank Accession Number
Barbula unguiculata	LC747006
Chorisodontium aciphyllum	MW355440
Dicranum hengduanensis	NC_080897
Didymodon constrictus	NC_069304
Fissidens crispulus I	**PX108640**
Fissidens crispulus II	**PX108641**
Fissidens nobilis	NC_044155
Fissidens protonematicola	LC761303
Hyophila propagulifera	LC747008
Hypopterygium fauriei	PQ774561
Leucobryum juniperoideum	MK952779
Pohlia cruda	NC_056136
Pseudocrossidium replicatum	NC_056241
Schistidium sp	MW429504
Sphagnum junghuhnianum	NC_060704
Sphagnum multifibrosum	NC_060705
Sphagnum riparium	PP626113
Sphagnum subsecundum	MW528203
Streblotrichum convolutum	LC747010
Syntrichia filaris	NC_050352
Syntrichia ruralis	NC_012052
Takakia lepidozioides	MZ895084
Tortula acaulon	PQ839656
Tortula atrovirens	PP190927
Weissia exserta	LC769575

Note: Accession numbers in bold represent sequences newly generated in this work.

Table S2. Functional annotation and classification of chloroplast genomes of *Fissidens crispulus*.

Category for genes	Group of genes	Name of genes
Genes for photosynthesis	Subunits of photosystem I	*psaA, psaB, psaC, psaI, psaJ, psaM*
	Subunits of photosystem II	*psbA, psbB, psbC, psbD, psbE, psbF, psbH, psbI, psbJ, psbK, psbL, psbM, psbN, psbT, psbZ*
	Subunits of	*ndhA*, ndhB*, ndhC, ndhD, ndhE,*

	NADH-dehydrogenase	*ndhF, ndhG, ndhH, ndhI, ndhJ, ndhK*
	Subunits of cytochrome b/f complex	*petA, petB*, petD*, petG, petL*
	Subunits of ATP synthase	*atpA, atpB, atpE, atpF*, atpH, atpI*
	Large subunit of rubisco	*rbcL*
	Genes related to chlorophyll biosynthesis	*chlL, chlN, chlB*
Self-replication	rRNA genes Large subunit of ribosome	*rpl2, rpl14, rpl16*, rpl20, rpl21, rpl22, rpl23, rpl32, rpl33, rpl36*
	Small subunit of ribosome	*rps2, rps3, rps4, rps7, rps8, rps11, rps12*, rps14, rps15, rps18, rps19*
	DNA dependent RNA polymerase	*rpoB, rpoC1*, rpoC2*
	rRNA genes	*rrn4.5S, rrn5S, rrnl6S*（2）, *rrn23S*（2）
	tRNA genes	*trnY-GUA, trnW-CCA, trnV-UAC, trnV-GAC**（2）, *trnT-UGU, trnT-GGU, trnS-UGA, trnS-GGA, trnS-GCU, trnR-UCU*, trnR-CCG, trnR-ACG*（2）, *trnQ-UUG, trnP-UGG, trnP-GGG, trnN-GUU*（2）, *trnM-CAU*（2）, *trnL-UAG, trnL-UAA*, trnL-CAA, trnK-UUU*, trnI-GAU**（2）, *trnH-GUG, trnG-UCC, trnfM-CAU, trnF-GAA, trnE-UUC, trnD-GUC, trnC-GCA, trnA-UGC*（2）
Other genes	Maturase	*matK*
	Protease	*clpP***
	Envelope membrane protein	*cemA*
	Subunit of Acetyl-CoA-carboxylase	*accD*
	Translation initiation factor gene	*infA*
Genes of unknown function	Open Reading Frames (ORF, ycf)	*ycfl, ycf2, ycf3**, ycf4*，*ycf12*，*ycf66*

Note: * only one intron gene; ** two intron genes; gene (2): indicates the presence of two copies of the gene.

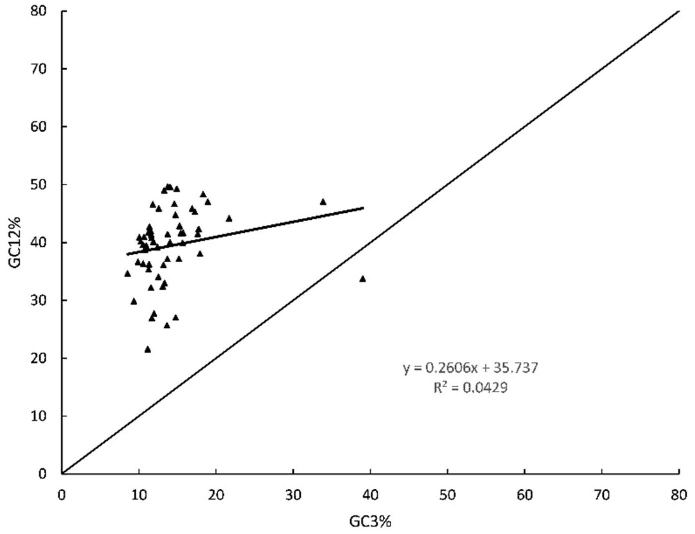

Figure S1. Neutrality plot analysis in *Fissidens crispulus* II. The correlations between the average GC codon content in GC1 and GC2 (GC12) and the third codon position (GC3) were analyzed and the standard curve and R^2 in *Fissidens crispulus* II, respectively. The X-axis represents GC3%, while the Y-axis represents GC12%.

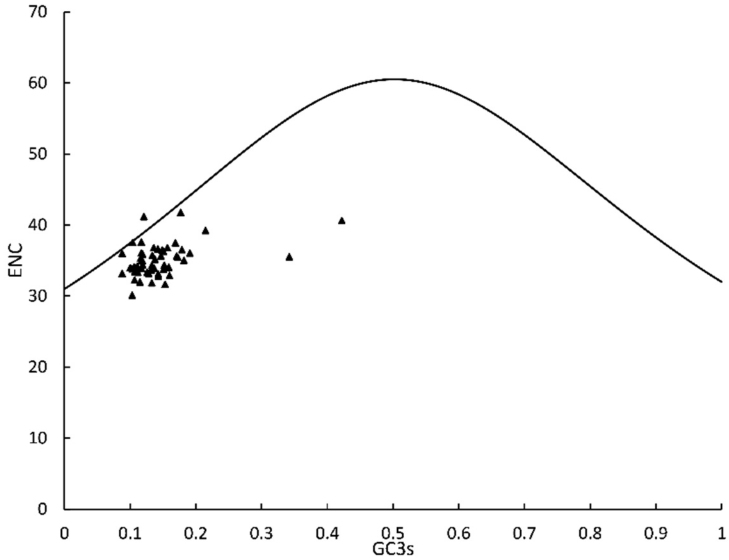

Figure S2. ENC-GC3 plot analysis in *Fissidens crispulus* II. The correlations between the effective number of codons (ENC) and the contents of the nucleotide G/C at the third codon synonymous location (GC3s) were analyzed in *Fissidens crispulus* II, respectively. The standard curve represents the functional relationship between ENC and GC3 under mutation pressure rather than natural selection. The X-axis represents GC3s, while the Y-axis represents ENC.

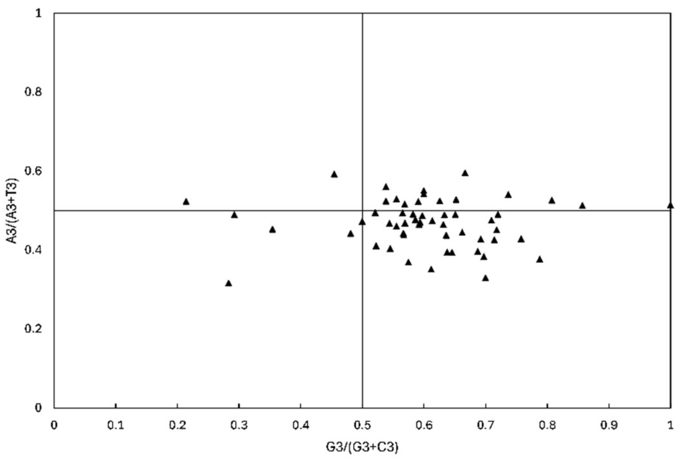

Figure S3. PR2-bias plot analysis in *Fissidens crispulus* II. The correlations between A3/(A3+U3) and G3/(G3+C3) were analyzed in *Fissidens crispulus* II, respectively. If the codon has no usage bias, A=T and C=G, the value was in the center point of the plot. The first quadrant represents the codon preference of A/G, and the third quadrant represents T/C preference. The X-axis represents G3/(G3+C3), while the Y-axis represents A3/(A3+U3).
